# Erratum to: ‘Complete genome sequence of Roseophage vB_DshP-R1, which infects Dinoroseobacter shibae DFL12’

**DOI:** 10.1186/s40793-015-0056-3

**Published:** 2015-09-24

**Authors:** Jianda Ji, Rui Zhang, Nianzhi Jiao

**Affiliations:** State Key Laboratory of Marine Environmental Science, Institute of Marine Microbes and Ecospheres, Xiamen University, Xiamen, PR, China

Unfortunately, the original version of this article [1] contained an error. The citation details was included incorrectly as 9:31. The correct citation is 10:6.
